# Modeling skin sensitization potential of mechanistically hard-to-be-classified aniline and phenol compounds with quantum mechanistic properties

**DOI:** 10.1186/2050-6511-15-76

**Published:** 2014-12-24

**Authors:** Qin Ouyang, Lirong Wang, Ying Mu, Xiang-Qun Xie

**Affiliations:** Department of Pharmaceutical Sciences, Computational Chemical Genomics Screening Center, School of Pharmacy, NIH National Center, of Excellence for Computational Drug Abuse Research, Drug Discovery Institute, Pittsburgh, PA 15261 USA; Department of Computational and Systems Biology, University of Pittsburgh, Pittsburgh, PA 15261 USA; Division of Biology, Office of Science and Engineering Laboratories, Center for Devices and Radiobiological Health, US Food and Drug Administration, Silver Spring, MD 20993 USA; College of Pharmacy, Third Military Medical University, Chongqing, 400038 China

**Keywords:** Chemical mechanisms, Structure-activity relationship, Skin sensitizer, Anilines, Phenols, Quantum mechanism

## Abstract

**Background:**

Advanced structure-activity relationship (SAR) modeling can be used as an alternative tool for identification of skin sensitizers and in improvement of the medical diagnosis and more effective practical measures to reduce the causative chemical exposures. It can also circumvent ethical concern of using animals in toxicological tests, and reduce time and cost. Compounds with aniline or phenol moieties represent two large classes of frequently skin sensitizing chemicals but exhibiting very variable, and difficult to predict, potency. The mechanisms of action are not well-understood.

**Methods:**

A group of mechanistically hard-to-be-classified aniline and phenol chemicals were collected. An *in silico* model was established by statistical analysis of quantum descriptors for the determination of the relationship between their chemical structures and skin sensitization potential. The sensitization mechanisms were investigated based on the features of the established model. Then the model was utilized to analyze a subset of FDA approved drugs containing aniline and/or phenol groups for prediction of their skin sensitization potential.

**Results and discussion:**

A linear discriminant model using the energy of the highest occupied molecular orbital (*ϵ*_HOMO_) as the descriptor yielded high prediction accuracy. The contribution of *ϵ*_HOMO_ as a major determinant may suggest that autoxidation or free radical binding could be involved. The model was further applied to predict allergic potential of a subset of FDA approved drugs containing aniline and/or phenol moiety. The predictions imply that similar mechanisms (autoxidation or free radical binding) may also play a role in the skin sensitization caused by these drugs.

**Conclusions:**

An accurate and simple quantum mechanistic model has been developed to predict the skin sensitization potential of mechanistically hard-to-be-classified aniline and phenol chemicals. The model could be useful for the skin sensitization potential predictions of a subset of FDA approved drugs.

**Electronic supplementary material:**

The online version of this article (doi:10.1186/2050-6511-15-76) contains supplementary material, which is available to authorized users.

## Background

Skin sensitization related dermatitis and rash represents the most common manifestation of chemical immunotoxicity in humans, which results in a cost estimated $1 billion annually due to lost work, reduced productivity, medical care, and disability payments in USA [1, 2]. In addition, as part of the regulatory review process, an increase in the incidence of skin allergies and hypersensitivity-related adverse events associated with the use of FDA regulated products or approved drugs has been observed, suggesting a safety gap between premarket review and the post market surveillance [2].

Common testing methods to assess skin sensitization potential of materials include: (1) guinea pig maximization test (GPMT); (2) murine-based local lymph node assay (LLNA). In GPMT tests, hazard identification is done by visual observations of erythema and edema reactions, which are subjective, are difficult to differentiate between contact allergens and strong irritants, and is time consuming [3]. The LLNA is recommended by international regulatory agencies; however, inconsistencies between LLNA and clinical observations have been documented [4]. Considering the existence of vast compounds around today, developing rapid and effective methods for chemical sensitizer identification/risk assessment is still a challenge [2].

*In silico* approaches are an attractive alternative to animal testing through analyzing the structural features of sensitizers/non-sensitizers to derive predictive rules or models [5]. The risks of thousands of commercially available chemicals could be assessed in a cost effective manner. Among these approaches, mechanism based rules, which investigate the structural characteristics of sensitizers, are promising [6].

Historically, the first study of chemical reactivity and skin sensitization was reported in 1936 [7]. A mechanism of small organic molecules to form an immunogenic complex by reacting with macromolecules (proteins or others) in the skin to cause sensitization was postulated. Currently, a more plausible mechanism reported involves a formation of covalent bonding between electrophilic allergens and nucleophilic moieties of amino acids from skin proteins (usually side chains) [8]. Such amino acids include cysteine thiol (mainly) and lysine (amino), and to a lesser extent arginine, histidine, methionine and tyrosine [9]. Based on the well-established principles of mechanistic organic chemistry, the skin sensitization potential of a chemical in many cases was predicted by its reactivity with these residues [9, 10]. However, some compounds need to be activated via either autoxidation outside the skin (prehaptens) or bioactivation inside the skin (prohaptens) to be able to form immunogenic complexes with skin proteins [11].

Structure-activity relationship (SAR) studies of skin sensitization potential have been successfully carried out for epoxyaldehydes [12], enone [13], halogenated aromatics [14], benzaldehydes [15], dienes [16], oximes [17], aldehydes [18] and epoxides [19]. Aniline/aromatic amine and/or phenol derivatives are two large classes of frequently sensitizing chemicals. Quite a few pilot studies have been conducted [20–23]. Roberts *et al.* specifically investigated the sensitization mechanisms of diaminobenzenes or dihydroxylbenzenes [24]. However, the predictability of the skin sensitization potential for these two classes of chemicals is unsatisfactory [6, 25]. Further exploration of novel sensitization mechanisms will be informative for constructing better SAR models/rules. In addition, aniline and phenol moieties that are often present in approved drugs can also cause skin sensitization. For example, contact dermatitis occurs in one individual following prolonged subcutaneous infusion of hydromorphone [26], a cancer pain treatment agent which contains one phenol moiety.

Drug-induced skin reactions may be associated with several biological mechanisms, but in many cases the precise mechanism is unclear [27]. It is well-known that Type IV allergic reaction induced by many chemicals and drugs is a T-cell mediated delay type hypersensitivity which can cause skin sensitization/dermatitis [27].

In this study, we intended to establish an *in silico* model for a class of mechanistically hard-to-classify anilines and phenols to study the relationship between their chemical reactivity and biological allergic response. We then investigated sensitization mechanisms of action associated with these compounds based on the features of this model. The model was further utilized to analyze a subset of FDA approved drugs containing aniline and/or phenol groups in skin sensitization potential. The predicted skin sensitization potential for these drugs was validated according to relevant literatures and adverse event reports.

## Methods

### Data sets

A data set of 63 chemicals, including 30 anilines and 33 phenols, was collected from published literature [11, 23, 28–36]. Chemicals with well-known allergic mechanisms, *i.e.* Michael acceptors (MA), SN_2_ electrophiles, S_N_Ar electrophiles, Schiff base formers, and acylation agents, were excluded from the data set. For example, pentachlorophenol (CAS: 87-86-5) belongs to S_N_Ar electrophiles; benzyl salicylate (CAS: 118-58-1) and 3,3′,4′,5-tetrachlorosalicylanilide (CAS: 1154-59-2) are acylation agents. In addition, chemicals having two OH and NH_2_ substituents at aromatic rings were also excluded from the data set because these compounds are known to easily form a benzoquinone (a Michael acceptor) or a nitrogen analogue of benzoquinone (also a Michael acceptor) [24]. Finally, a list of 30 chemicals was obtained (Table 1). They represent a class of mechanistically hard-to-be-classified compounds because they can’t be classified into any of the abovementioned five categories. These compounds were then randomly split into a training set of 15 compounds and a test set of 15 compounds. As shown in Table 1, the training set includes 7 anilines and 8 phenols, while the test set includes 6 anilines and 9 phenols. The detailed information including initial screening of the 63 selected chemicals is available as Additional file 1.

**Table 1:** **Summary of the** ***ϵ***_**HOMO**_ **, predicted values of the 30 chemicals and their experimentally determined data** [11, 23, 28–36]

ID	Cas #	Name	Sensitizer	EC3	Ref.	***ϵ***_HOMO_(hartree)	***P***
1	101-80-4	4,4-diaminodiphenylether	Y		30	-0.262	1.0694
2	106-47-8	4-Chloroaniline	Y	6.5	33,35	-0.289	0.6563
3	150-13-0	4-Aminobenzoic acid	N		31	-0.306	0.3962
4	369-36-8	2-Fluoro-5-nitroaniline	N		34	-0.325	0.1055
5	538-41-0	4,4-diaminoazobenzene	Y		30	-0.258	1.1306
6	62-53-3	Aniline	Y	89	31,36	-0.286	0.7022
7	79456-26-1	3-Chloro-5-(trifluoromethyl)-2-pyridinamine	N		23	-0.326	0.0902
8	2050-14-8	2,2′-Azodiphenol	Y	27.9	34	-0.294	0.5798
9	55845-90-4	(N-Benzyl-N-ethylamino)-3′-hydroxyacetophenone hydrochloride	N		23	-0.316	0.2432
10	69-72-7	Salicylic acid	N		31	-0.343	-0.1699
11	90-15-3	1-Naphthol	Y	1.3	11,29.33	-0.274	0.8858
12	94-13-3	Propylparaben	N		11	-0.329	0.0443
13	97-54-1	Isoeugenol	Y	3.5	31,33,32	-0.277	0.8399
14	99-96-7	4-Hydrobenzoic acid	N		11	-0.341	-0.1393
15	186743-26-0	3-Methyleugenol	Y	32	33,36	-0.294	0.5798
16	101-77-9*	4,4-diaminodiphenylmethane	Y		30	-0.266	1.0082
17	121-57-3*	Sulphanilic acid	N		11	-0.322	0.1514
18	537-65-5*	4,4-diaminodiphenylamine	Y		30	-0.243	1.3601
19	60-09-3*	4-aminophenylazobenzene	Y		30	-0.276	0.8552
20	63-74-1*	Sulfanilamide	N		11	-0.307	0.3809
21	94-09-7*	Benzocaine	N		33	-0.303	0.4421
22	15128-82-2*	3-Hydroxy-2-nitropyridine	N		31	-0.362	-0.4606
23	2785-87-7*	Dihydroeugenol	Y	12.45	11,29,32	-0.288	0.6716
24	619-14-7*	3-Hydroxy-4-nitrobenzoic acid	N		31	-0.371	-0.5983
25	80-05-7*	bisphenol A	Y		30	-0.287	0.6869
26	93-51-6*	2-Methoxy-4-methyl-phenol	Y	5.8	11,29,32	-0.288	0.6716
27	97-53-0*	Eugenol	Y	13.95	31,32,33	-0.296	0.5492
28	99-76-3*	Methyl 4-hydroxybenzoate	N		11	-0.329	0.0443
29	119-36-8*	Methyl salicylate	N		28	-0.326	0.0902
30	831-82-3*	4-Phenoxyphenol	Y		33	-0.293	0.5951

*Test set.

### Quantum mechanics calculations

All chemical optimization and subsequent orbital analysis were performed by using the Gaussian 03 suite of programs [37]. Chemicals were optimized using the AM1 Hamiltonian with the default optimization criteria [38, 39]. Calculations of the frontier molecular orbital, charge distribution and other quantum properties were carried out by using the 6-31Gd basis set. The quantum descriptors used in this study include the energies of the highest occupied molecular orbital (*ϵ*_HOMO_), the lowest unoccupied molecular orbital (*ϵ*_LUMO_), the second lowest unoccupied molecular orbital (*ϵ*_LUMO+1_), the second highest occupied molecular orbital (*ϵ*_HOMO-1_), the Mulliken atomic charges of the most negative (*Q*_min_) and most positive atoms (*Q*_max_), the Mulliken atomic charges of the N atom (*Q*_N_) in anilines or O atom (*Q*_O_) in phenols, the average of the absolute values of the charges on all atoms (*Q*_m_), and molecular dipole moment (*μ*). The shapes of the resulting orbitals were visualized using the GaussView application within Gaussian 03. All structures were either drawn or converted from SMILES (Simplified molecular-input line-entry system) strings, using Chembiodraw Ultra V12.0 (PerkinElmer Informatics Desktop Software).

### Statistical analysis

The skin sensitization potency of a compound was symbolized by 1 (Yes) and 0 (No). The values of each quantum descriptor were linearly normalized to the same range (0 to 1), stepwise linear regressions between the quantum properties and experimental outputs of the training set were performed by the statistical package of R program version 3.0.0 [40]. The properties with lower weighting factors were abandoned in the second step of linear regression.

## Results and discussion

The compounds with aniline and/or phenol moieties can be classified into a single subclass for consideration of skin sensitizers. However, not all of the compounds possessing aniline or phenol groups are sensitizers, suggesting some compounds can form covalent bonds with skin proteins whereas others cannot. In this study, the sensitization potential of anilines and phenols were modeled using quantum mechanical descriptors.

### Modeling the skin sensitization potential by quantum properties of anilines and phenols

The coefficient constant of *ϵ*_HOMO_ was determined as the highest weighting factor based on the results of linear regression analysis. The skin sensitization potential of anilines and phenols can be formulated as:
1

**Figure 1:**
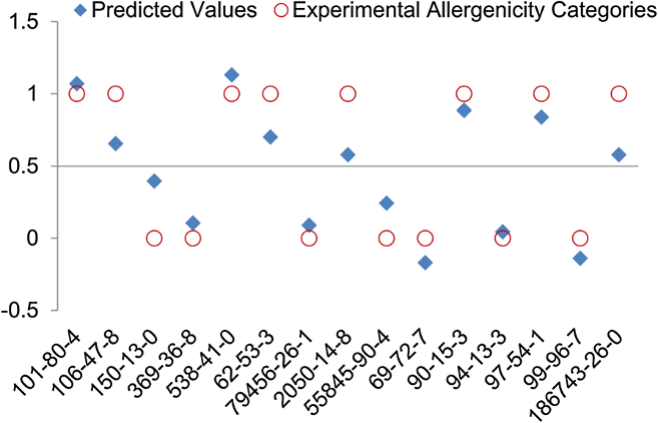
**Correlation of skin sensitization potential of anilines and phenols in the training set between experimental allergenicity categories and predicted values from the model built with quantum mechanistic properties.** Experimental allergenicity categories: 1 for sensitizer and 0 for non-sensitizer respectively; Predicted Value (*P*) = 15.30 *ϵ_HOMO_ + 5.08. A compound with a *P* greater than 0.50 is predicted as a sensitizer; otherwise, it is predicted as a non-sensitizer.

**Figure 2:**
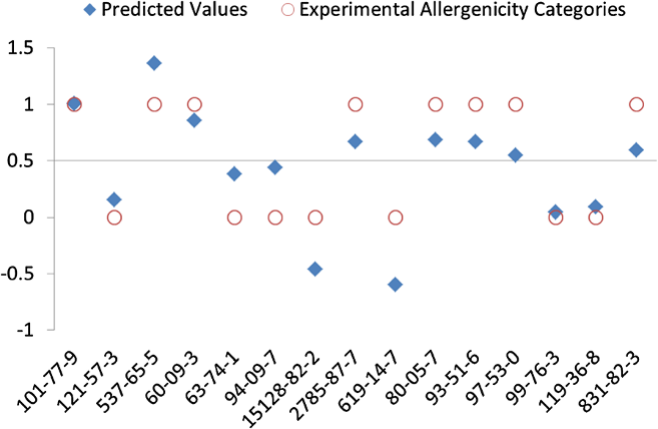
**Correlation of skin sensitization potential of anilines and phenols in the test set between experimental allergenicity categories and predicted values from the model built with quantum mechanistic properties.** Experimental allergenicity categories: 1 for sensitizer and 0 for non-sensitizer respectively; Predicted Value (*P*) = 15.30 *ϵ_HOMO_ + 5.08. A compound with the *P* greater than 0.50 is predicted as a sensitizer; otherwise, it is predicted as a non-sensitizer.

The median of the symbolized skin sensitization potency, 0.50, was considered as the threshold for prediction of sensitizers and non-sensitizers. An aniline or phenol is predicted as a sensitizer if *P* is greater than 0.50, and as a non-sensitizer if *P* is below 0.5. With a threshold of P =0.50, Equation 1 implies that a chemical within the applicability domain is predicted to be a skin sensitizer if the HOMO energy is greater than -0.30 Hartree ((0.5-5.08)/15.30 = -0.299 ≈ -0.30). The experimental allergenicity categories (sensitizer or non-sensitizer) and predicted results of the training set are shown in Figure 1, where red-open circle dots indicate well-known sensitizers at 1 and non-sensitizers at 0, respectively. The blue-solid-diamond dots indicate the predicted values. All of the training compounds were correctly predicted by Formula 1. The same model was applied to the test set. Interestingly, all test compounds were correctly predicted (Figure 2). The total prediction accuracy of chemicals in training and test sets was 100% (30/30). The model shows very high accuracy and only depends on the value of *ϵ*_HOMO_, suggesting that *ϵ*_HOMO_ is a key factor for the assessment of skin sensitization potential of those aniline and phenol containing compounds.

The linear relationship between the predicted values and *ϵ*_HOMO_ also suggests that a chemical with higher predicted value implies a higher reactivity for oxidation consequently resulting in higher skin sensitization potential. The LLNA data as a quantitative endpoint, posed a semi-dose-dependent manner, allows for prediction of potency. The EC3 values (effective concentration for a three-fold proliferation of lymph node cells) from the reported LLNA experiments of most allergen phenols were also collected as shown in Table 1. Weak sensitizers with higher EC3 values (meaning lower sensitization potential) have smaller *P* values. For example, *P* values of five weak sensitizers with100 > EC3 > 10 *i.e.,* eugenol (CAS: 97-53-0, EC3 = 13.95), Dihydroeugenol (CAS: 2785-87-7, EC3 = 12.45), 2,2’-azodiphenol (CAS: 2050-14-8, EC3 = 27.90), 3-methyleugenol (CAS: 186743-26-0,EC3 = 32), and aniline (CAS: 62-53-3, EC3 = 89) were 0.549, 0.672, 0.580, 0.580, and 0.702, respectively. On the other hand, two moderate sensitizers, 2-Methoxy-4-methyl-phenol (CAS: 93-51-6, EC3 = 5.8), 4-Chloroaniline (CAS: 106-47-8, EC3 = 6.5), have a slightly higher *P* value 0.672, 0.656, respectively. Another two moderate sensitizers with smaller EC3 value, isoeugenol (CAS: 97-54-1, EC3 = 3.5) and 1-naphthol (CAS: 90-15-3, EC3 = 1.3) have greater *P* values, 0.840 and 0.886, respectively. For most chemicals, their -logEC3 values correlate with *P* values quite well, but for aniline, its –logEC3 value is much less potent than its *P* value predicted. This may indicate that the initial oxidation of aniline, which is quite fast, is not in this case the rate-determining step for protein haptenation. The analysis of the relationship between EC3 and ϵ_HOMO_ for these nine chemicals was reported in the Additional file 1.

### Possible reaction mechanisms of aromatic anilines and phenols

Occurrence of electrophilic–nucleophilic reactions between chemical and skin proteins is a primary reason of chemical induced skin sensitization [8]. Most chemicals with high skin sensitization potential can be classified as Michael acceptors (MA), SN_2_ electrophiles, S_N_Ar electrophiles, Schiff base formers, or acylation agents. The reaction mechanisms of anilines and phenols, however, are poorly understood and very few of them can be classified into the aforementioned five categories. One proposed mechanism is that sensitization occurs via oxygen attack ortho to an amino group or via oxidative quinone-methide formation [25, 41]. For example, Roberts *et al.* reported the mechanistic chemistry of aromatic diamino-, dihydroxy-, and amino-hydroxy compounds [24] where two parallel chemical mechanisms were described as the most possible processes: oxidation to electrophilic (protein reactive) quinones, quinone imines, or quinone di-imines or formation of protein reactive free radicals. These mechanisms, unfortunately, are not applicable to the all single NH/OH substituted anilines and phenols. For instance, aniline and 4-butylaniline are sensitizers whereas 4-aminobenzoic acid, 4-aminobenzenesulfonamide, and 4-aminobenzenesulfonic acid are non-sensitizers. Beside the solubility effects and the formation of ions/zwitterions, the reactivity variety of chemical entities by substituent effects play an important role in reducing dermal penetration and immunogenicity of protein conjugates.

By analyzing the relationship between quantum properties and chemical reactivity, we successfully modeled the skin sensitization potential of two groups of chemicals (aromatic anilines and phenols) with a single coefficient of *ϵ*_HOMO_, while the energy of the lowest unoccupied molecular orbital (*ϵ*_LUMO_), considered as the critical factor for most electrophilic reactions [8, 11], was poorly correlated with sensitization potential. These results suggest the skin sensitization mechanism of those compounds may result from several steps but not a directly electrophilic reaction.

**Scheme 1:**
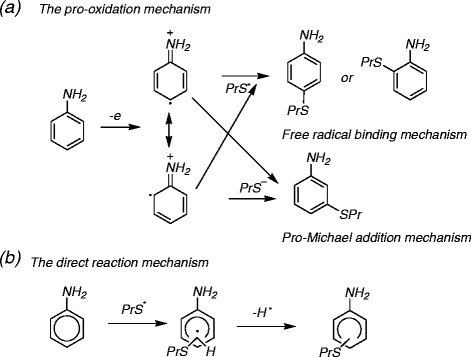
Reaction mechanisms of anilines binding to protein **Reaction mechanisms of anilines binding to protein. (a)** The pro-oxidation mechanism. **(b)** The direct reaction mechanism.

The *ϵ*_HOMO_ dependent results implied that a process of losing electron may be involved in the activation of those sensitizers. Those compounds may be activated via an autoxidation mechanism to further interact with skin proteins as prehaptens. In addition, the mechanisms where these chemicals directly react with free radical of skin proteins also should be considered [42]. In the present study, we proposed that two potential pathways could lead these compounds to cause skin sensitization as shown in Scheme 1 [42]. In the first pathway (Scheme 1a), an aniline (or a phenol) is readily oxidized to a radical cation through loss of an electron at the aromatic ring [43] and forms two possible reactive intermediates. A protein-associated sulfhydryl radical then attacks the aromatic of the radical cation to form a covalent bond at the orth- or para-position. Or, the reactive intermediates bind to nucleophilic moieties on proteins through the Michael addition. The second pathway corresponds to what Lepoittevin defined as a direct haptenation route [44], whereby attack of a protein associated sulfhydryl radical on the ring gives an intermediate radical (Scheme 1b).

**Figure 3:**
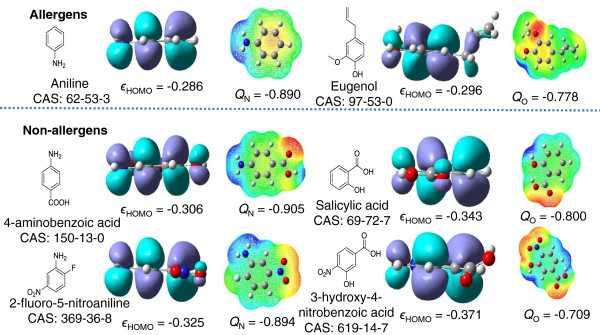
**The structures, energies and shapes of HOMO, and charge density of representative anilines and phenols.** Anilines (left column: aniline, 4-aminobenzoic acid, and 2-fluoro-5-nitroaniline); Phenols (right column: eugenol, salicylic acid, and 3-hydroxy-4-nitrobenzoic acid). The unit of the energy is hartree.

A compound with lower energy of HOMO appears either more stable or less reactive when reacting with a protein associated sulfhydryl radical [24]. Therefore, as shown in Figure 3, the sensitizers *e.g.,* the aniline (CAS: 62-53-3) and the eugenol (CAS: 97-53-0) equipped with higher *ϵ*_HOMO_ values can lose electron(s) more easily to form radical cation intermediates than non-sensitizers (e.g., 2-fluoro-5-nitroaniline (CAS: 369-36-8), 4-aminobenzoic acid (CAS: 150-13-0), salicylic acid (CAS: 69-72-7), and 3-hydroxy-4-nitrobenzoic acid (CAS: 619-14-7)). We noted that the non-sensitizers of anilines and phenols are those that have the electron withdrawing groups attached to the aromatic ring, such as –F, -NO_2_, -COOH. This implies that introduction of electron withdrawing groups to the aromatic ring of anilines or phenols may be one of the effective ways to reduce the potency of skin sensitizers.

### Predicting skin sensitization potentials of a subset of FDA approved drugs with aniline and phenol groups

There are no effective tools to predict the skin sensitization potential of drugs, because drug-induced skin reactions may be caused by several mechanisms either single or mixed [27]. The skin sensitization, in the context, refers to T-cell mediated sensitization (type IV allergy). The reaction of chemicals with proteins was recognized as one of the necessary process of the T-cell mediated sensitization [23]. The *in silico* mechanistic models may offer valuable insights into better understanding the initiation of drug induced allergies.

We collected 53 drugs containing aniline and/or phenol moieties from the DrugBank database. The information of these 53 compounds is also available in the Additional file 1. These FDA approved drugs were then analyzed to filter out those with structural alerts of skin sensitization. The sulfonamide drugs were also removed due to they have different mechanisms of action. For example, sulfamethoxazole (DrugBank ID: DB01015) can be oxidized to a hydroxylamine metabolite and subsequently form a reactive nitroso intermediate by auto-oxidation that enables it to react with skin proteins [45]. Finally, twenty six compounds were obtained and their skin sensitization potential was predicted by our model. Among these 26 compounds, six of them were reported to be able to induce “allergic dermatitis” according to the side effect information in MetaADEDB database (Table 2). Interestingly, our results showed that five of them, e.g. Clenbuterol, Dapsone, Morphine, Hydromorphone and Raloxifene were correctly predicted as sensitizers (Table 2) as their *P* values are greater than the threshold 0.50. In addition, allergic dermatitis is a rare side effect of Liothyronine according to the information from https://www.universaldrugstore.com/medications/Liothyronine/side-effects. However, users should be cautious that there is no label for drugs not causing “allergic dermatitis”, thus it is hard to find a negative set in FDA approved drugs to further evaluate our model.

**Table 2:** **Prediction of skin sensitization potential for 6 FDA approved drugs that have side effect of allergic dermatitis reported in MetaADEDB database**

	DrugBank ID	Name	***P***	Prediction ^a^	MetaADEDB ^***b***^
1	DB00279	Liothyronine	-0.001	N	Y
2	DB01407	Clenbuterol	0.589	Y	Y
3	DB00250	Dapsone	0.700	Y	Y
4	DB00295	Morphine	0.580	Y	Y
5	DB00327	Hydromorphone	0.595	Y	Y
6	DB00481	Raloxifene	0.977	Y	Y

^*a*^A drug is predicted as an sensitizer if its *P* value is greater than 0.50; Otherwise, as a non-sensitizer. ^*b*^Compounds having the keywords “allergic dermatitis” in their side effect reports in the MetaADEDB database are indicated as sensitizers. *Y*: Sensitizer; *N*: Non-sensitizer.

## Conclusion

This study has demonstrated how quantum chemical calculations can be utilized to predict skin sensitization potential and to infer the reaction mechanism for a class of mechanistically hard-to-be-classified chemicals containing aniline and phenol moieties. The outcomes emphasized that the energy of highest occupied molecular orbital plays an important role for predicting skin sensitization potential of these compounds, indicating the activation process occurred via either autoxidation or direct reaction with free radical. Our model was further applied to predict the allergenic potential of the approved drugs containing aniline and/or phenol moieties. Several of these drugs were identified as sensitizers and the prediction agreed well with their “allergic dermatitis” side effect. Thus, the data indicate that our newly developed *in silico* algorithm shows promise as a preclinical risk assessment tool for screening allergenic potential.

Again, we should point out that skin allergic reactions are not commonly seen for drugs given via the oral route. Though they may share similar mechanisms, caution should be taken when extrapolating our model from skin sensitization potential for topically applied chemicals to predict “allergic potential” of drugs.

## Electronic supplementary material

Additional file 1: **Predicted values and experimental data of reported chemicals and FDA approved drugs that contain aniline and/or phenol moieties.** (DOCX 394 KB)

## Abbreviations

ACD: Allergic contact dermatitis
AM1: Austin model 1
CAS: Chemical abstracts service
EC3: Effective concentration for a three-fold proliferation of lymph node cells
FDA: Food and drug administration
GPMT: Guinea pig maximization test
HOMO: Highest occupied molecular orbital
LLNA: The murine-based local lymph node assay
LUMO: Lowest unoccupied molecular orbital
MA: Michael acceptors
SAR: Structure-activity relationship
SMILES: Simplified molecular-input line-entry system
SN_2_: A kind of nucleophilic substitution reaction mechanism
S_N_Ar: Nucleophilic aromatic substitution.
