# Supplementary material for: Modeling skin sensitization potential of mechanistically hard-to-be-classified aniline and phenol compounds with quantum mechanistic properties
Source: BMC Pharmacol Toxicol. 2014 Dec 24;15:76. doi: 10.1186/2050-6511-15-76 (PMC4298069; doi:10.1186/2050-6511-15-76)
Supplement: Supplementary file 1 — Additional file 1: Predicted values and experimental data of reported chemicals and FDA approved drugs that contain aniline and/or phenol moieties. (DOCX 394 KB) [file 40360_2014_361_MOESM1_ESM.docx]

Supplementary Information for:

# Modeling Skin Sensitization Potential of Mechanistically Hard-to-be-Classified Aniline and Phenol Compounds with Quantum Mechanistic Properties

Qin Ouyang^1.3#^, Lirong Wang^1#^, Ying Mu^2^ and Xiang-Qun Xie^1^*

^1^ Department of Pharmaceutical Sciences, Computational Chemical Genomics Screening Center, School of Pharmacy; Pittsburgh Chemical Methods and Library Development Center; Drug Discovery Institute, and Department of Computational Biology, University of Pittsburgh, Pittsburgh, PA 15261, USA.

^2^ Division of Biology, Office of Science and Engineering Laboratories, Center for Devices and Radiobiological Health, US Food and Drug Administration, Silver Spring, MD 20993, USA.

^3^College of Pharmacy, Third Military Medical University, Chongqing 400038, China

^#^ Authors with equal contributions

Qin Ouyang: [ouyangq@gmail.com](mailto:ouyangq@gmail.com)

Lirong Wang: [liw30@pitt.edu](mailto:liw30@pitt.edu)

Ying Mu: [ying.mu@fda.hhs.gov](mailto:ying.mu@fda.hhs.gov)

*Corresponding author: Xiang-Qun (Sean) Xie, [xix15@pitt.edu](mailto:xix15@pitt.edu) ; Tel.: +1-412-383-5276; Fax: +1-412-383-7436

Table 1. Summary of the Calculated Reactivity Parameters, Predicted Values, and Experimentally Determined Data for 63 Chemicals[[1-11](#_ENREF_1)]. ( ID 1-30 : Substituted Aniline, ID 31-63 : Substituted Phenols)

| ID | Cas # | Name | SMILES*^a^* | Sensitizer*^b^* | EC3 | Reference | *ϵ*_LUMO_  (hartree) | *ϵ*_HOMO_  (hartree) | *P* |
| --- | --- | --- | --- | --- | --- | --- | --- | --- | --- |
| 1 | 100-63-0 | phenylhydrazine | NNC1=CC=CC=C1 | Y |  | 1 | 0.152 | -0.288 | 0.672 |
| 2 | 101-54-2 | 4-aminodiphenylamine | NC1=CC=C(C=C1)NC2=CC=CC=C2 | Y |  | 1 | 0.139 | -0.260 | 1.100 |
| 3 | 101-77-9 | 4,4-diaminodiphenylmethane | NC1=CC=C(C=C1)CC2=CC=C(N)C=C2 | Y |  | 1 | 0.139 | -0.266 | 1.008 |
| 4 | 101-80-4 | 4,4-diaminodiphenylether | NC1=CC=C(C=C1)OC2=CC=C(N)C=C2 | Y |  | 1 | 0.132 | -0.262 | 1.069 |
| 5 | 104147-32-2 | 3,5-Dichloro-4-(1,1,2,2-tetrafluoroethoxy)aniline | NC1=CC(Cl)=C(OC(F)(F)C(F)F)C(Cl)=C1 | N |  |  | 0.105 | -0.316 | 0.243 |
| 6 | 106-50-3 | p-phenylenediamine | NC1=CC=C(N)C=C1 | Y | 0.29 | 1/7/9 | 0.147 | -0.254 | 1.192 |
| 7 | 108-45-2 | 3-Phenylenediamine | NC1=CC=CC(N)=C1 | Y | 0.49 | 6/9 | 0.160 | -0.273 | 0.901 |
| 8 | 121-57-3 | Sulphanilic acid | O=S(O)(C(C=C1)=CC=C1N)=O | N |  | 6 | 0.098 | -0.322 | 0.151 |
| 9 | 123-30-8 | p-aminophenol | NC1=CC=C(O)C=C1 | Y |  | 1 | 0.140 | -0.271 | 0.932 |
| 10 | 150-13-0 | 4-Aminobenzoic acid | OC(C1=CC=C(N)C=C1)=O | N |  | 5 | 0.101 | -0.306 | 0.396 |
| 11 | 20048-27-5 | Bandrowski's base | NC1=CC=C(/N=C2C=C(N)/C(C=C\2N)=N/C3=CC=C(N)C=C3)C=C1 | Y | 0.04 | 2 | 0.059 | -0.251 | 1.238 |
| 12 | 2835-95-2 | 5-Amino-2-methylphenol | OC1=CC(N)=CC=C1C | Y | 3.4 | 2 | 0.154 | -0.275 | 0.871 |
| 13 | 2835-99-6 | 4-amino-3-methyl-phenol | NC1=CC=C(O)C=C1C | Y | 1.45 | 2 | 0.144 | -0.267 | 0.993 |
| 14 | 2871-01-4 | 2-(4-Amino-2-nitrophenylamino)-ethanol | NC1=CC=C(NCCO)C([N+]([O-])=O)=C1 | Y | 2.2 | 6/7/9 | 0.061 | -0.284 | 0.733 |
| 15 | 369-36-8 | 2-Fluoro-5-nitroaniline | NC1=CC([N+]([O-])=O)=CC=C1F | N |  | 2 | 0.056 | -0.325 | 0.106 |
| 16 | 5307-14-2 | 2-nitro-p-phenylenediamine | O=[N+]([O-])C1=CC(N)=CC=C1N | Y | 0.4 | 1/7/9 | 0.066 | -0.279 | 0.809 |
| 17 | 537-65-5 | 4,4-diaminodiphenylamine | NC1=CC=C(C=C1)NC(C=C2)=CC=C2N | Y |  | 1 | 0.140 | -0.243 | 1.360 |
| 18 | 538-41-0 | 4,4-diaminoazobenzene | NC(C=C1)=CC=C1/N=N/C2=CC=C(N)C=C2 | Y |  | 1 | 0.084 | -0.258 | 1.131 |
| 19 | 591-27-5 | 3-Aminophenol | OC1=CC=CC(N)=C1 | Y |  | 4/9/6 | 0.152 | -0.289 | 0.656 |
| 20 | 60-09-3 | 4-aminophenylazobenzene | NC(C=C1)=CC=C1/N=N/C2=CC=CC=C2 | Y |  | 1 | 0.074 | -0.276 | 0.855 |
| 21 | 610-81-1 | 4-Amino-3-nitrophenol | NC1=CC=C(O)C=C1[N+]([O-])=O | Y | 0.2 | 2 | 0.060 | -0.297 | 0.534 |
| 22 | 62-53-3 | Aniline | NC1=CC=CC=C1 | Y | 89 | 5,11 | 0.149 | -0.286 | 0.702 |
| 23 | 6358-09-4 | 2-Amino-6-chloro-4-nitrophenol | NC1=CC([N+]([O-])=O)=CC(Cl)=C1O | Y | 6.85 | 2/9 | 0.058 | -0.322 | 0.151 |
| 24 | 63-74-1 | Sulfanilamide | O=S(N)(C(C=C1)=CC=C1N)=O | N |  | 6 | 0.122 | -0.307 | 0.381 |
| 25 | 79456-26-1 | 3-Chloro-5-(trifluoromethyl)-2-pyridinamine | NC1=NC=C(C(F)(F)F)C=C1Cl | N |  | 3 | 0.093 | -0.326 | 0.090 |
| 26 | 82560-06-3 | 3-(1-Ethyl-1-methylpropyl)-5-isoxazolamine | NC1=CC(C(C)(CC)CC)=NO1 | N |  | 7 | 0.162 | -0.313 | 0.289 |
| 27 | 95-55-6 | 2-Aminophenol | OC1=CC=CC=C1N | Y | 0.5 | 6/7/8/9 | 0.149 | -0.280 | 0.794 |
| 28 | 99-56-9 | 4-Nitro-1,2-benzenediamine | NC1=CC=C([N+]([O-])=O)C=C1N | Y | 0.05 | 2 | 0.073 | -0.306 | 0.396 |
| 29 | 106-47-8*^c^* | 4-Chloroaniline | NC1=CC=C(Cl)C=C1 | Y* |  | 8,10 | 0.137 | -0.289 | 0.656 |
| 30 | 94-09-7 | Benzocaine | O=C(c1ccc(N)cc1)OCC | N |  | 8 | 0.105 | -0.303 | 0.442 |
| 31 | 1166-52-5 | Lauryl gallate | O=C(C1=CC(O)=C(O)C(O)=C1)OCCCCCCCCCCCC | Y | 0.3 | 4/9 | 0.090 | -0.322 | 0.151 |
| 32 | 10597-60-1 | Hydroxytyrosol | OC1=CC=C(CCO)C=C1O | Y | 0.6 | 2 | 0.136 | -0.305 | 0.412 |
| 33 | 108-46-3 | Resorcinol | OC1=CC=CC(O)=C1 | Y* |  | 1/9 | 0.144 | -0.307 | 0.381 |
| 34 | 1154-59-2 | 3,3',4',5-Tetrachlorosalicylanilide | OC1=C(Cl)C=C(Cl)C=C1C(NC2=CC=C(Cl)C(Cl)=C2)=O | Y |  | 1 | 0.065 | -0.322 | 0.151 |
| 35 | 118-58-1 | Benzyl salicylate | O=C(C1=CC=CC=C1O)OCC2=CC=CC=C2 | Y | 2.9 | 2 | 0.081 | -0.323 | 0.136 |
| 36 | 121-32-4 | Ethyl vanillin | O=CC1=CC=C(O)C(OCC)=C1 | N |  | 6 | 0.083 | -0.317 | 0.228 |
| 37 | 121-33-5 | Vanillin | OC1=CC=C(C=O)C=C1OC | N |  | 6 | 0.085 | -0.313 | 0.289 |
| 38 | 123-31-9 | 1,4-Hydroquinone | OC1=CC=C(O)C=C1 | Y | 0.1 | 4/9 | 0.131 | -0.291 | 0.626 |
| 39 | 150-75-4 | 4-(Methylamino)phenol sulphate | OC1=CC=C(NC)C=C1 | Y |  | 6 | 0.143 | -0.269 | 0.962 |
| 40 | 15128-82-2 | 3-Hydroxy-2-nitropyridine | OC1=CC=CN=C1[N+]([O-])=O | N |  | 2 | 0.041 | -0.362 | -0.461 |
| 41 | 1776-30-3 | 2',4'-Dihydroxychalcone | O=C(C1=CC=C(O)C=C1O)/C=C/C2=CC=CC=C2 | Y | 0.56 | 2 | 0.051 | -0.312 | 0.304 |
| 42 | 2050-14-8 | 2,2'-Azodiphenol | OC1=CC=CC=C1/N=N/C2=CC=CC=C2O | Y | 27.9 | 2 | 0.053 | -0.294 | 0.580 |
| 43 | 2657-25-2 | 4'-Hydroxychalcone | O=C(C1=CC=C(O)C=C1)/C=C/C2=CC=CC=C2 | Y | 0.002 | 2 | 0.056 | -0.310 | 0.335 |
| 44 | 2785-87-7 | Dihydroeugenol | COC1=CC(CCC)=CC=C1O | Y | 12.45 | 6/7/9 | 0.148 | -0.288 | 0.672 |
| 45 | 526-37-4 | atranol | O=Cc1c(O)cc(cc1O)C | Y | 0.6 | 2 | 0.081 | -0.316 | 0.243 |
| 46 | 55302-96-0 | 2-Methyl-5-hydroxyethylaminophenol | CC1=CC=C(NCCO)C=C1O | Y | 0.4 | 6/7/9 | 0.160 | -0.283 | 0.748 |
| 47 | 55845-90-4 | (N-Benzyl-N-ethylamino)-3'-hydroxyacetophenone hydrochloride | O=C(C1=CC=CC(O)=C1)CN(CC2=CC=CC=C2)CC.Cl | N |  | 3 | 0.085 | -0.316 | 0.243 |
| 48 | 619-14-7 | 3-Hydroxy-4-nitrobenzoic acid | O=C(C(C=C1O)=CC=C1[N+]([O-])=O)O | N |  | 2 | 0.011 | -0.371 | -0.598 |
| 49 | 65235-31-6 | 3-Nitro-N-(2-hydroxyethyl)-4-aminophenol | OC1=CC=C(NCCO)C([N+]([O-])=O)=C1 | Y | 0.07 | 2 | 0.109 | -0.300 | 0.488 |
| 50 | 69-72-7 | Salicylic acid | OC1=CC=CC=C1C(O)=O | N |  | 5 | 0.011 | -0.343 | -0.170 |
| 51 | 80-05-7 | bisphenol A | CC(C1=CC=C(O)C=C1)(C)C2=CC=C(O)C=C2 | Y |  | 1 | 0.132 | -0.287 | 0.687 |
| 52 | 87-86-5 | Pentachlorophenol | ClC1=C(O)C(Cl)=C(Cl)C(Cl)=C1Cl | Y |  | 5 | 0.077 | -0.341 | -0.139 |
| 53 | 90-15-3 | 1-Naphthol | OC1=CC=CC2=CC=CC=C21 | Y | 1.3 | 6/7/8 | 0.104 | -0.274 | 0.886 |
| 54 | 93-51-6 | 2-Methoxy-4-methyl-phenol | COC1=CC(C)=CC=C1O | Y | 5.8 | 6/7/8/9 | 0.146 | -0.288 | 0.672 |
| 55 | 94-13-3 | Propylparaben | O=C(C1=CC=C(O)C=C1)OCCC | N |  | 6 | 0.096 | -0.329 | 0.044 |
| 56 | 97-53-0 | Eugenol | OC1=CC=C(CC=C)C=C1OC | Y | 13.95 | 5/7/8/9 | 0.137 | -0.296 | 0.549 |
| 57 | 97-54-1 | Isoeugenol | OC1=CC=C(/C=C/C)C=C1OC | Y | 3.5 | 5/7/8/9 | 0.120 | -0.277 | 0.840 |
| 58 | 98-29-3 | p-tert-butylcatechol | OC1=CC(C(C)(C)C)=CC=C1O | Y |  | 1 | 0.141 | -0.293 | 0.595 |
| 59 | 99-76-3 | Methyl 4-hydroxybenzoate | OC1=CC=C(C(OC)=O)C=C1 | N |  | 6 | 0.096 | -0.329 | 0.044 |
| 60 | 99-96-7 | 4-Hydrobenzoic acid | OC1=CC=C(C(O)=O)C=C1 | N |  | 6 | 0.092 | -0.341 | -0.139 |
| 61 | 119-36-8 | Methyl salicylate | OC1=CC=CC=C1C(OC)=O | N |  | 4 | 0.086 | -0.326 | 0.090 |
| 62 | 186743-26-0 | 3-Methyleugenol | OC1=C(C)C=C(CC=C)C=C1OC | Y | 32 | 8,11 | 0.143 | -0.294 | 0.580 |
| 63 | 831-82-3 | 4-Phenoxyphenol | OC1=CC=C(OC2=CC=CC=C2)C=C1 | Y |  | 8 | 0.124 | -0.293 | 0.595 |

*^a^*The SMILES was generated from Chembiodraw Ultra V12.0 (PerkinElmer Informatics Desktop Software). *^b^*All the Y/N correspond to experimental data from the references cited. *^c^*In the ref 8, 4-Chloroaniline was reported as no-sensitizer.

Table 2. Summary of the Predicted Value and MetaADEDB Data for 53 FDA Approved Drugs.

|  | DrugBank ID | Name | SMILES*^d^* | *P* | Other allergic group^a^ | Sulfonamide Derivative*^b^* | MetaADEDB*^c^* |
| --- | --- | --- | --- | --- | --- | --- | --- |
| 1 | DB01298 | Sulfacytine | CCN1C=CC(NS(=O)(=O)C2=CC=C(N)C=C2)=NC1=O | 0.202 | N | Y |  |
| 2 | DB06288 | Amisulpride | O=C(NCC1N(CC)CCC1)c2cc(c(N)cc2(OC))S(=O)(=O)CC | 0.222 | N | Y |  |
| 3 | DB06150 | Sulfadimethoxine | O=S(=O)(Nc1nc(nc(OC)c1)OC)c2ccc(N)cc2 | 0.297 | N | Y |  |
| 4 | DB00701 | Amprenavir | CC(C)CN(C[C@@H](O)[C@H](CC1=CC=CC=C1)NC(=O)O[C@H]1CCOC1)S(=O)(=O)C1=CC=C(N)C=C1 | 0.308 | N | Y | Y |
| 5 | DB01581 | Sulfamerazine | CC1=NC(NS(=O)(=O)C2=CC=C(N)C=C2)=NC=C1 | 0.348 | N | Y |  |
| 6 | DB01582 | Sulfamethazine | CC1=CC(C)=NC(NS(=O)(=O)C2=CC=C(N)C=C2)=N1 | 0.369 | N | Y |  |
| 7 | DB00576 | Sulfamethizole | CC1=NN=C(NS(=O)(=O)C2=CC=C(N)C=C2)S1 | 0.38 | N | Y |  |
| 8 | DB00259 | Sulfanilamide | NC1=CC=C(C=C1)S(N)(=O)=O | 0.383 | N | Y |  |
| 9 | DB01015 | Sulfamethoxazole | CC1=CC(NS(=O)(=O)C2=CC=C(N)C=C2)=NO1 | 0.384 | N | Y | Y |
| 10 | DB01299 | Sulfadoxine | COC1=NC=NC(NS(=O)(=O)C2=CC=C(N)C=C2)=C1OC | 0.389 | N | Y |  |
| 11 | DB00891 | Sulfapyridine | NC1=CC=C(C=C1)S(=O)(=O)NC1=CC=CC=N1 | 0.4 | N | Y |  |
| 12 | DB06147 | Sulfathiazole | O=S(=O)(Nc1nccs1)c2ccc(N)cc2 | 0.415 | N | Y |  |
| 13 | DB01264 | Darunavir | [H][C@@]12CCO[C@]1([H])OC[C@@H]2OC(=O)N[C@@H](CC1=CC=CC=C1)[C@H](O)CN(CC(C)C)S(=O)(=O)C1=CC=C(N)C=C1 | 0.427 | N | Y | Y |
| 14 | DB00263 | Sulfisoxazole | CC1=NOC(NS(=O)(=O)C2=CC=C(N)C=C2)=C1C | 0.448 | N | Y |  |
| 15 | DB00664 | Sulfametopyrazine | COC1=NC=CN=C1NS(=O)(=O)C1=CC=C(N)C=C1 | 0.466 | N | Y |  |
| 16 | DB08798 | Sulfamoxole | CC1=C(C)N=C(NS(=O)(=O)C2=CC=C(N)C=C2)O1 | 0.718 | N | Y |  |
| **17** | **DB00250** | **Dapsone** | **NC1=CC=C(C=C1)S(=O)(=O)C1=CC=C(N)C=C1** | **0.7** | **N** |  | **Y** |
| **18** | **DB00509** | **Dextrothyroxine** | **N[C@H](CC1=CC(I)=C(OC2=CC(I)=C(O)C(I)=C2)C(I)=C1)C(O)=O** | **-0.093** | **N** |  |  |
| **19** | **DB08810** | **Cinitapride** | **O=C(NC1CCN(CC1)CC2CC=CCC2)c3cc(c(N)cc3(OCC))[N+](=O)[O-]** | **0.169** | **N** |  |  |
| **20** | **DB00279** | **Liothyronine** | **N[C@@H](CC1=CC(I)=C(OC2=CC(I)=C(O)C=C2)C(I)=C1)C(O)=O** | **-0.001** | **N** |  | **Y** |
| **21** | **DB01161** | **Chloroprocaine** | **CCN(CC)CCOC(=O)C1=C(Cl)C=C(N)C=C1** | **0.306** | **N** |  |  |
| **22** | **DB00721** | **Procaine** | **CCN(CC)CCOC(=O)C1=CC=C(N)C=C1** | **0.435** | **N** |  |  |
| **23** | **DB01086** | **Benzocaine** | **CCOC(=O)C1=CC=C(N)C=C1** | **0.442** | **N** |  |  |
| **24** | **DB00135** | **L-Tyrosine** | **N[C@@H](CC1=CC=C(O)C=C1)C(O)=O** | **0.274** | **N** |  |  |
| **25** | **DB00388** | **Phenylephrine** | **CNC[C@H](O)C1=CC(O)=CC=C1** | **0.274** | **N** |  |  |
| **26** | **DB00892** | **Oxybuprocaine** | **CCCCOC1=C(N)C=CC(=C1)C(=O)OCCN(CC)CC** | **0.482** | **N** |  |  |
| **27** | **DB01233** | **Metoclopramide** | **CCN(CC)CCNC(=O)C1=CC(Cl)=C(N)C=C1OC** | **0.487** | **N** |  |  |
| **28** | **DB00604** | **Cisapride** | **CO[C@H]1CN(CCCOC2=CC=C(F)C=C2)CC[C@H]1NC(=O)C1=CC(Cl)=C(N)C=C1OC** | **0.489** | **N** |  |  |
| **29** | **DB00807** | **Proparacaine** | **CCCOC1=C(N)C=C(C=C1)C(=O)OCCN(CC)CC** | **0.519** | **N** |  |  |
| **30** | **DB00963** | **Bromfenac** | **NC1=C(CC(O)=O)C=CC=C1C(=O)C1=CC=C(Br)C=C1** | **0.552** | **N** |  |  |
| **31** | **DB01035** | **Procainamide** | **CCN(CC)CCNC(=O)C1=CC=C(N)C=C1** | **0.561** | **N** |  |  |
| **32** | **DB00486** | **Nabilone** | **[H][C@@]12CC(=O)CC[C@@]1([H])C(C)(C)OC1=CC(=CC(O)=C21)C(C)(C)CCCCCC** | **0.411** | **N** |  |  |
| **33** | **DB01407** | **Clenbuterol** | **CC(C)(C)NCC(O)C1=CC(Cl)=C(N)C(Cl)=C1** | **0.589** | **N** |  | **Y** |
| **34** | **DB06802** | **Nepafenac** | **O=C(N)Cc2cccc(C(=O)c1ccccc1)c2(N)** | **0.683** | **N** |  |  |
| **35** | **DB00470** | **Dronabinol** | **[H][C@@]12C=C(C)CC[C@@]1([H])C(C)(C)OC1=CC(CCCCC)=CC(O)=C21** | **0.549** | **N** |  |  |
| **36** | **DB00295** | **Morphine** | **[H][C@@]12OC3=C(O)C=CC4=C3[C@@]11CCN(C)[C@]([H])(C4)[C@]1([H])C=C[C@@H]2O** | **0.580** | **N** |  | **Y** |
| **37** | **DB00327** | **Hydromorphone** | **[H][C@@]12OC3=C(O)C=CC4=C3[C@@]11CCN(C)[C@]([H])(C4)[C@]1([H])CCC2=O** | **0.595** | **N** |  | **Y** |
| **38** | **DB00504** | **Levallorphan** | **[H][C@@]12CCCC[C@@]11CCN(CC=C)[C@@H]2CC2=C1C=C(O)C=C2** | **0.611** | **N** |  |  |
| **39** | **DB00913** | **Anileridine** | **CCOC(=O)C1(CCN(CCC2=CC=C(N)C=C2)CC1)C1=CC=CC=C1** | **0.825** | **N** |  |  |
| **40** | **DB00163** | **Vitamin E** | **CC(C)CCC[C@@H](C)CCC[C@@H](C)CCC[C@]1(C)CCC2=C(O1)C(C)=C(C)C(O)=C2C** | **0.855** | **N** |  |  |
| **41** | **DB00481** | **Raloxifene** | **OC1=CC=C(C=C1)C1=C(C(=O)C2=CC=C(OCCN3CCCCC3)C=C2)C2=C(S1)C=C(O)C=C2** | **0.977** | **N** |  | **Y** |
| **42** | **DB01123** | **Proflavine** | **NC1=CC2=NC3=C(C=CC(N)=C3)C=C2C=C1** | **1.092** | **N** |  |  |
| 43 | DB00359 | Sulfadiazine | NC1=CC=C(C=C1)S(=O)(=O)NC1=NC=CC=N1 | 0.339 | Y | Y | Y |
| 44 | DB00233 | Aminosalicylic Acid | NC1=CC(O)=C(C=C1)C(O)=O | 0.321 | Y |  |  |
| 45 | DB00480 | Lenalidomide | NC1=CC=CC2=C1CN(C1CCC(=O)NC1=O)C2=O | 0.405 | Y |  | Y |
| 46 | DB00345 | Aminohippurate | O=C(O)CNC(=O)c1ccc(N)cc1 | 0.427 | Y |  |  |
| 47 | DB00522 | Bentiromide | OC(=O)C1=CC=C(NC(=O)[C@H](CC2=CC=C(O)C=C2)NC(=O)C2=CC=CC=C2)C=C1 | 0.32 | Y |  |  |
| 48 | DB00357 | Aminoglutethimide | CCC1(CCC(=O)NC1=O)C1=CC=C(N)C=C1 | 0.573 | Y |  |  |
| 49 | DB00964 | Apraclonidine | NC1=CC(Cl)=C(NC2=NCCN2)C(Cl)=C1 | 0.576 | Y |  |  |
| 50 | DB01208 | Sparfloxacin | C[C@H]1CN(C[C@@H](C)N1)C1=C(F)C(N)=C2C(=O)C(=CN(C3CC3)C2=C1F)C(O)=O | 0.638 | Y |  |  |
| 51 | DB00244 | Mesalazine | NC1=CC(C(O)=O)=C(O)C=C1 | 0.695 | Y |  |  |
| 52 | DB00316 | Acetaminophen | CC(=O)NC1=CC=C(O)C=C1 | 0.748 | Y |  | Y |
| 53 | DB00255 | Diethylstilbestrol | CCC(=C(CC)C1=CC=C(O)C=C1)C1=CC=C(O)C=C1 | 0.794 | Y |  |  |

*^a^* Y means the drug: (1) has group( s) to form a SN2 electrophile, SNAr electrophile, Schiff base former, and acylation agent, and Michael receptor ; or (2) has two OH and NH2 substituents at aromatic rings. Other drugs are labeled as N. *^b^* Y means the compounds are sulfonamide derivatives.*^c^* Y means the keywords “allergic dermatitis” can be found in the drug’s side effect report from MetaADEDB. *^d^* Y means the keywords “allergic dermatitis” can be found in the drug’s side effect report from MetaADEDB.

**The correlation between EC3 Values and ϵ_HOMO_**

The LLNA data as a quantitative endpoint, posed a semi-dose-dependent manner, allows for modeling of potency. The EC3 values from the reported LLNA experiments of 9 chemicals were also collected as shown in Table 1. The calculated energy of HOMO (ϵ_HOMO_) was further employed for prediction the EC3 values of these 9 chemicals. The final linear regression model is shown in Formula S1 and the plot of the correlation between experimental and predicted pEC3 were shown in **Figure S1A**.

pEC3 = -13.16 – 49.44* ϵ_HOMO_ (Hartree) (S1)

For most chemicals, their -logEC3 values correlate with P values quite well, but for aniline, its –logEC3 value is much less potent than its P value predicted. This may indicate that the initial oxidation of aniline, which is quite fast, is not in this case the rate-determining step for protein haptenation. Excluding aniline results in a significantly improved model for the remaining 8 chemicals (Formula S2 and **Figure S1B**).

pEC3 = -14.21 – 52.67 * ϵ_HOMO_ (Hartree) (S2)


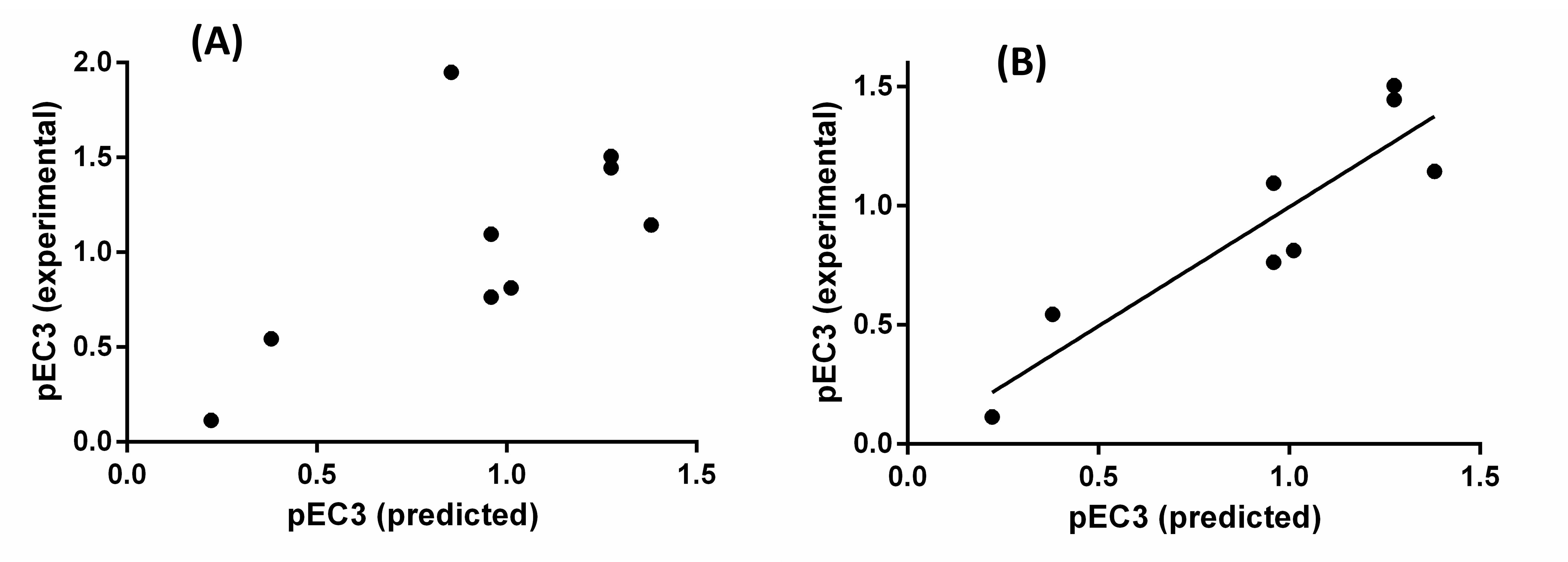


**Figure S1. The correlation between experimental (y-axis) and predicted (x-axis) pEC3 values of 9 chemicals using ϵ_HOMO_ as the model descriptor(A. considering all of the data; B. excluding aniline).**

References:

1. Barratt MD, Langowski JJ: **Validation and subsequent development of the DEREK skin sensitization rulebase by analysis of the BgVV list of contact allergens**. *Journal of Chemical Information and Computer Sciences* 1999, **39**(2):294-298.

2. Kern PS, Gerberick GF, Ryan CA, Kimber I, Aptula A, Basketter DA: **Local Lymph Node Data for the Evaluation of Skin Sensitization Alternatives: A Second Compilation**. *Dermatitis* 2010, **21**(1):8-32.

3. Payne MP, Walsh PT: **Structure-Activity-Relationships for Skin Sensitization Potential - Development of Structural Alerts for Use in Knowledge-Based Toxicity Prediction Systems**. *Journal of Chemical Information and Computer Sciences* 1994, **34**(1):154-161.

4. Gerberick GF, Vassallo JD, Bailey RE, Chaney JG, Morrall SW, Lepoittevin JP: **Development of a peptide reactivity assay for screening contact allergens**. *Toxicological Sciences* 2004, **81**(2):332-343.

5. Schneider K, Akkan Z: **Quantitative relationship between the local lymph node assay and human skin sensitization assays**. *Regulatory Toxicology and Pharmacology* 2004, **39**(3):245-255.

6. Enoch SJ, Madden JC, Cronin MTD: **Identification of mechanisms of toxic action for skin sensitisation using a SMARTS pattern based approach**. *Sar and Qsar in Environmental Research* 2008, **19**(5-6):555-578.

7. Estrada E, Patlewicz G, Gutierrez Y: **From knowledge generation to knowledge archive. a general strategy using TOPS-MODE with DEREK to formulate new alerts for skin Sensitization**. *Journal of Chemical Information and Computer Sciences* 2004, **44**(2):688-698.

8. Miller MD, Yourtee DM, Glaros AG, Chappelow CC, Eick JD, Holder AJ: **Quantum mechanical structure-activity relationship analyses for skin sensitization**. *Journal of Chemical Information and Modeling* 2005, **45**(4):924-929.

9. Roberts DW, Patlewicz G, Kern PS, Gerberick F, Kimber I, Dearman RJ, Ryan CA, Basketter DA, Aptula AO: **Mechanistic applicability domain classification of a local lymph node assay dataset for skin sensitization**. *Chemical Research in Toxicology* 2007, **20**(7):1019-1030.

10. Estrada E, Patlewicz G, Chamberlain M, Basketter D, Larbey S: **Computer-aided knowledge generation for understanding skin sensitization mechanisms: The TOPS-MODE approach**. *Chemical Research in Toxicology* 2003, **16**(10):1226-1235.

11. Basketter DA: **Skin sensitization: strategies for the assessment and management of risk**. *British Journal of Dermatology* 2008, **159**(2):267-273.
